# Can adaptive clinical trials help to solve the productivity crisis of the pharmaceutical industry? - a scenario analysis

**DOI:** 10.1186/s13561-021-00302-6

**Published:** 2021-01-16

**Authors:** Jörg Mahlich, Arne Bartol, Srirangan Dheban

**Affiliations:** 1 Health Economics and Outcomes Research, Janssen, Pharmaceutical Companies of Johnson & Johnson, Neuss, Germany; 2 Düsseldorf Institute of Competition Economics (DICE), University of Düsseldorf, Düsseldorf, Germany; 3 Government Affairs, Janssen, Pharmaceutical Companies of Johnson & Johnson, Neuss, Germany

## Abstract

**Aim:**

The productivity of pharmaceutical research and development (R&D) investments is declining due to high failure rates in clinical research. Recently, the US Food and Drug Administration (FDA) acknowledged that adaptive designs can make drug development more efficient and less costly. Our objective is to simulate cost-saving effects and estimate the impact on global R&D expenditures as well as possible outcomes measured in life-years gained.

**Methods:**

Based on published drug-development cost data we calculate potential cost savings derived from variations in clinical success rates that result from employing adaptive trial designs. In a subsequent step we estimate how those cost changes affect global R&D expenditures and outcomes.

**Results:**

Our calculations indicate that an adaptive trial design with the potential to increase success rates of clinical trials by 4 percentage points could lower development costs for a new drug from 2.6 to 2.2bn USD. On a global scale, this cost reduction would free up an additional 4.2bn USD for investment into pharmaceutical R&D to bring about drug innovations that in turn would be capable of generating up to 3.5 million life-years.

**Conclusion:**

New clinical trial designs are crucial to improving productivity within the pharmaceutical industry and to fostering a sustainable health-care system.

## Introduction

The increasing health care expenditure as share of the Gross Domestic Product (GDP) has been subject to considerable political debate. The Organisation for Economic Co-Operation and Development (OECD) concluded in a report that health-care costs in advanced economies are rising so fast as to become unaffordable by mid-century if there are no reforms [1]. It was the economist William Baumol who coined the term “cost disease” when he offered the explanation that the increases in health-care spending can be attributed – at least in part- to pitfalls in productivity [2]. The theory predicts that rising productivity in manufacturing industries induces cost increases in labour-intensive services such as health care. This is because the health-care sector needs to raise wages by the same rates as the entire economy to attract talent and retain labour. As long as the price elasticity of demand is low, cost increases can be passed on to customers and patients. This allows for wage increases without causing unemployment although there are no productivity gains. Empirical economists have subsequently validated the theory and observed that health-care costs grow more rapidly when economy-wide wage increases exceed productivity gains [3, 4]. Although the pharmaceutical industry as part of the health-care system does not belong to the service sector, productivity decline is as big an issue for this industry as it is for the health-care sector in general. A study found that the number of new drugs approved per billion US dollars spent on R&D has halved roughly every 9 years since 1950, which means a drop by a factor of 80 in inflation-adjusted terms. The trend of falling R&D productivity in the pharmaceutical industry has been termed `Eroom’s Law’ in contrast to `Moore’s law’ that describes the productivity leaps in the semiconductor industry (in fact, it is `Moore’s Law’ read backwards) [5]. It was argued that low R&D productivity would put the entire business model of the pharmaceutical industry at risk. As Paul et al. [6] put it: “Without a substantial increase in R&D productivity, the pharmaceutical industry’s survival (let alone its continued growth prospects), at least in its current form, is in great jeopardy.” Paul et al. believe that a cost reduction of 50% per new chemical entity (NCE) will be needed to sustain a viable business model.

In parallel to the decreasing productivity, returns of investments in the pharmaceutical industry have dropped, a recent study found, analysing global stock market data from 1987 to 2012 [7]. In the second half of the observation period European pharmaceutical firms could not even earn their cost of capital and were actually destroying economic value, i.e. it would have been more efficient for European pharmaceutical companies to cut down on their investments and pay the money to their shareholders instead.

Some industry observers foresee a better R&D productivity in the future due to innovation and new technologies such as digitalisation. On the other hand, evidence suggests that the new technologies of the 1970’s and 1980’s such as computers and other IT devices have not necessarily contributed to increased productivity. Economics Nobel laureate Robert Solow once stated in a famous aphorism that “you can see the computer age everywhere but in the productivity statistics“ [8]. This so-called Solow paradox has been firmly supported with empirical studies that find little evidence of faster productivity growth in IT-intensive industries [9]. Economists like Scott Stern from MIT believe that general-purpose technologies take a lifetime to reorganize around and to show any measurable impact [10]. Hence, at least in the short and medium run, it is not so much new technology that will eventually reduce the cost of pharmaceutical R&D but rather new ways of running clinical trials, because one of the reasons for this declining productivity trend are ever increasing failure rates in clinical research, especially in phase III [11, 12].

The FDA was the first regulatory authority that recognized the need for modernising drug development to ensure future R&D investments: not only to accommodate rapid medical progress but also the limits to funding clinical trial programs. With respect to scientific progress, understanding the aetiology and pathogenesis of diseases at molecular level has led to a finer subdivision or differentiation of diseases and affected patients through the advent of biomarkers and the development of targeted drug therapies, resulting in precision medicine. As a consequence, it is becoming increasingly difficult to conduct classical confirmatory phase III studies as randomised controlled trials (RCT) with many hundreds of patients. One of the concepts under discussion, adaptive study design, enables biomarker-based hypotheses to be tested properly and comprehensively in clinical trials. This is because an adaptive study design enables prospectively planned interim evaluations that allow for modifications of the design during the course of the trial.

Later, in November 2019, the FDA published a remarkable guideline titled “Adaptive Designs for Clinical Trials of Drugs and Biologics”. Possible prospectively planned modifications to the study design include, for example, adaptations of the patient population, adaptations of a treatment arm, adaptations of patient allocation, or adaptations of endpoint selection [13]. The FDA (and subsequently also the European Medicines Agency [EMA]) has created a regulatory framework for evaluating adaptive study designs in the drug approval process [14], with the clear goal of making drug development more efficient. As the former FDA commissioner Scott Gottlieb phrased it: “Using more modern approaches to clinical trials, we can lower the cost of developing new drugs” [15]. The deployment of novel clinical trial designs incorporating adaptive components has the potential to not only reduce direct costs caused by stopping early due to futility but also reduce attrition rates due to modifications to the design during the course of a trial.

In contrast to fixed conventional designs, adaptive designs allow the prospectively planned modification of a clinical trial design using accumulated data. These modifications can involve one or more aspects of the trial design. In many cases ethical considerations as well as the economic burden for the companies are the most prominent reasons to use adaptive designs. In our particular case, we would like to look at the benefit of adaptive trial designs in terms of reducing the attrition rate. One of the most common adaptations is the sample size re-estimation after an interim analysis. In case the extent of a treatment effect is smaller than anticipated during the planning stage but still large enough to achieve clinical relevance, an adequate power can be achieved using such re-assessment procedures. This would reduce the attrition rates, as the sample size re-estimation would enable to show a treatment effect of statistical significance at the end of the study which may be slightly lower than anticipated, but still clinically meaningful. The reduction in attrition rates, however, will not be achieved by statistical significance only. The sample size re-assessment will only ensure enough statistical power at the end of the study for a smaller but still clinically meaningful treatment effect observed at the interim analysis (time-point of sample size re-assessment). Hence, re-estimating the sample size assumes that the magnitude of the effect seen at the interim analysis will remain until the end of the study. If a smaller effect size is shown at the end of the study compared to what was anticipated at the interim analysis, this effect size might not even be any longer clinically meaningful. In this case the sample size re-assessment will not lead a reduction of attrition rates. Other possibilities are adaptations of patient populations or treatment arms. An example for an adaptation of treatment arms is the STAMPEDE trial [16]. The STAMPEDE trial simultaneously evaluated multiple treatments for prostate cancer compared to a common control group. The design allowed for multiple interim analyses, with the option to terminate treatment arms that did not perform better compared to the common comparator. This design made simultaneous evaluation of multiple treatments more efficient than in multiple studies with fixed designs. Adaptation of a patient population make sense when there is some evidence that patients who belong to a certain sub-population experience a larger treatment effect than the overall population. Such sub-populations could be defined based on characteristics like demographics or genetic markers. A non-adaptive design would not allow restricting the enrollment to a targeted, more promising sub-population where treatment effects after a certain interim analysis are concerned. An adaptive design, on the other hand, allows such modifications and hence avoids negative trial results, although it has the potential of detecting a larger treatment effect in a certain sub-population. Such designs are often referred to as adaptive enrichment designs. It is worth mentioning that the concepts under discussion can in principle be translated into a Bayesian version as well which allows for borrowing of information from external sources and better decision making by forming trial success criteria such as concepts of probability of success [17].

Our aim in this study is to provide some estimates about the productivity effects of adaptive trial designs with respect to costs, R&D expenditures, and outcomes.

## Materials and methods

### Transition probabilities and pharmaceutical Research and Development costs

**Fig. 1:**
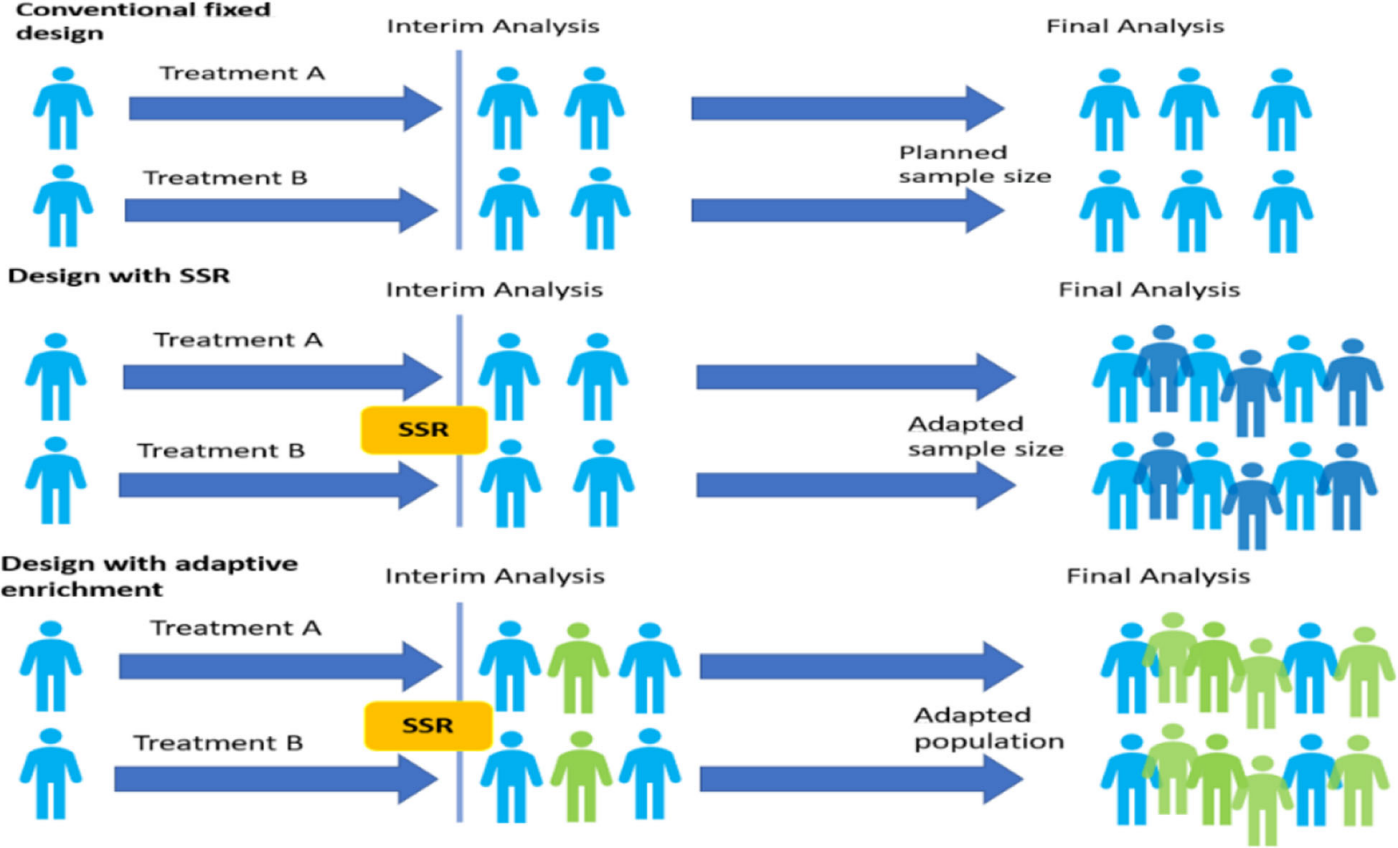
Schematic comparison of conventional fixed design and adaptive design with sample size re-assessment (SSR) as well as adaptive enrichment, adapted from Thorlund et al. (2018)

Clinical trials can fail for many reasons. These reasons can be: a lack of efficacy, safety issues, problems with patient recruitment, enrollment, and retention, or a lack of funding [18]. Specifically, for phase III, a recent review of 640 trials with novel therapeutics found that 54% failed during clinical development, with 57% of those failing due to inadequate efficacy [19]. We will simulate how variations in phase-III attrition rates caused by an alternative (i.e. adaptive) trial design may influence R&D cost for a new compound. An overview on the concepts of adaptive designs can be found in Thorlund et al. and Cerqueira et al. [20, 21]. Those concepts are visualized in Fig. 1.

**Fig. 2:**
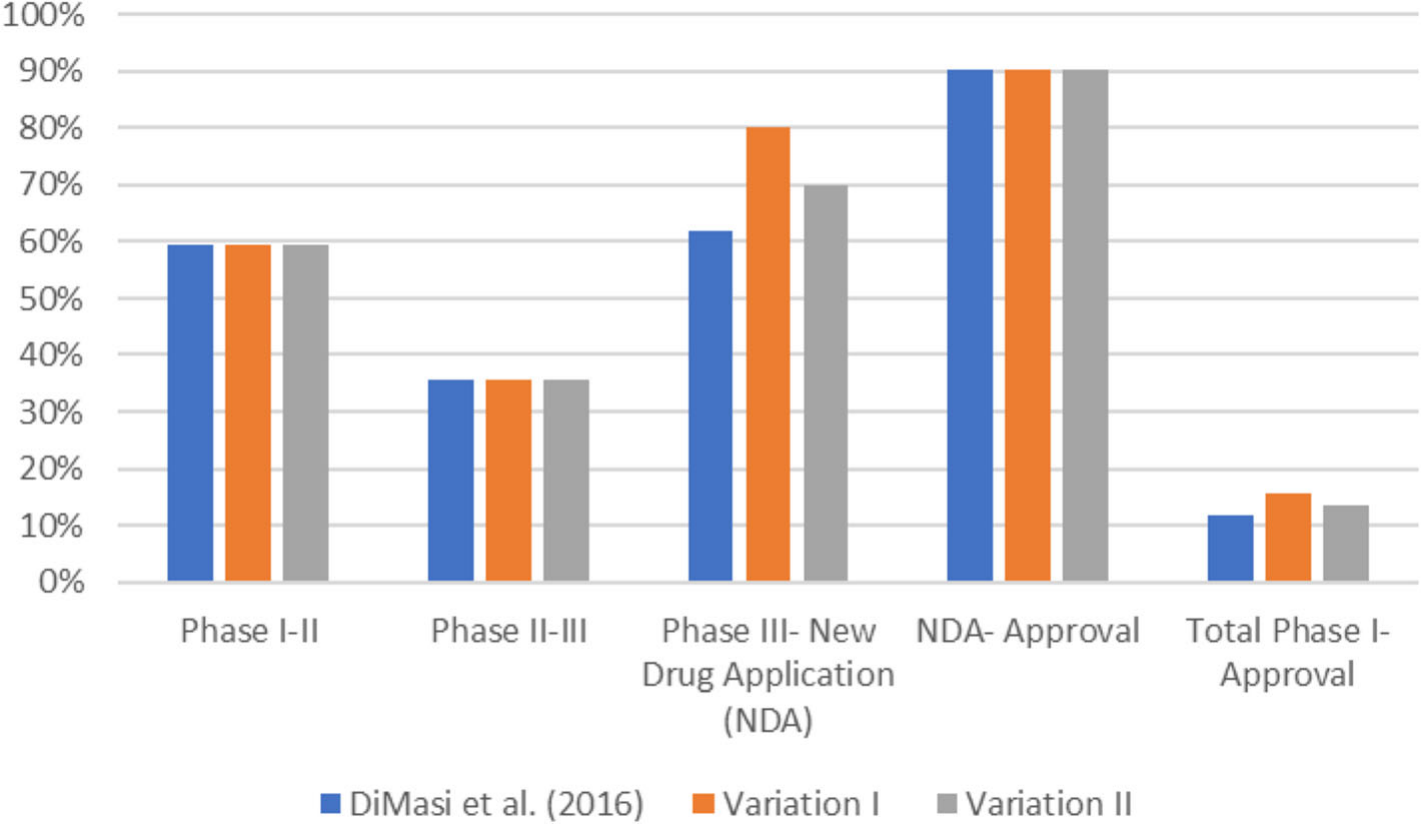
Phase specific transition probabilities [in %]

We will then provide some estimates on the impact on pharmaceutical R&D investments. Based on a study by DiMasi et al. on drug costs [22] a success rate of 63% in phase III is achieved. We hypothesise that using adaptive designs can lead to phase III success rates between 70 and 80%. This seems realistic as especially the option of sample size re-estimation offers the potential to decrease attrition rates due to the possibility to recruit more patients in case the effect seen in the study is smaller than anticipated. Survey results of the DIA Adaptive Design Scientific Working Group (ADSWG) show an increasing deployment of adaptive designs, leading to Marketing Authorizations which stresses also the acceptance of adaptive designs [23]. The selected gain in success rates should reflect a range of plausible (17%) (variation I) to conservative (7%) (variation II) gains in phase III success rates to allow an examination whether the results are robust. A comparison of the underlying transition probabilities stipulated by DiMasi et al. and ours are shown in Fig. 2. Total clinical success rates over all phases would rise from 11.83% (59,52% × 35.52% × 61.95% × 90.35% × 11.83%) to 15.81% (59,52% × 35.52% × 80.00% × 90.35% × 11.83%) in variation I and 13.37% (59,52% × 35.52% × 70.00% × 90.35% × 11.83%) in variation II.

**Fig. 3:**
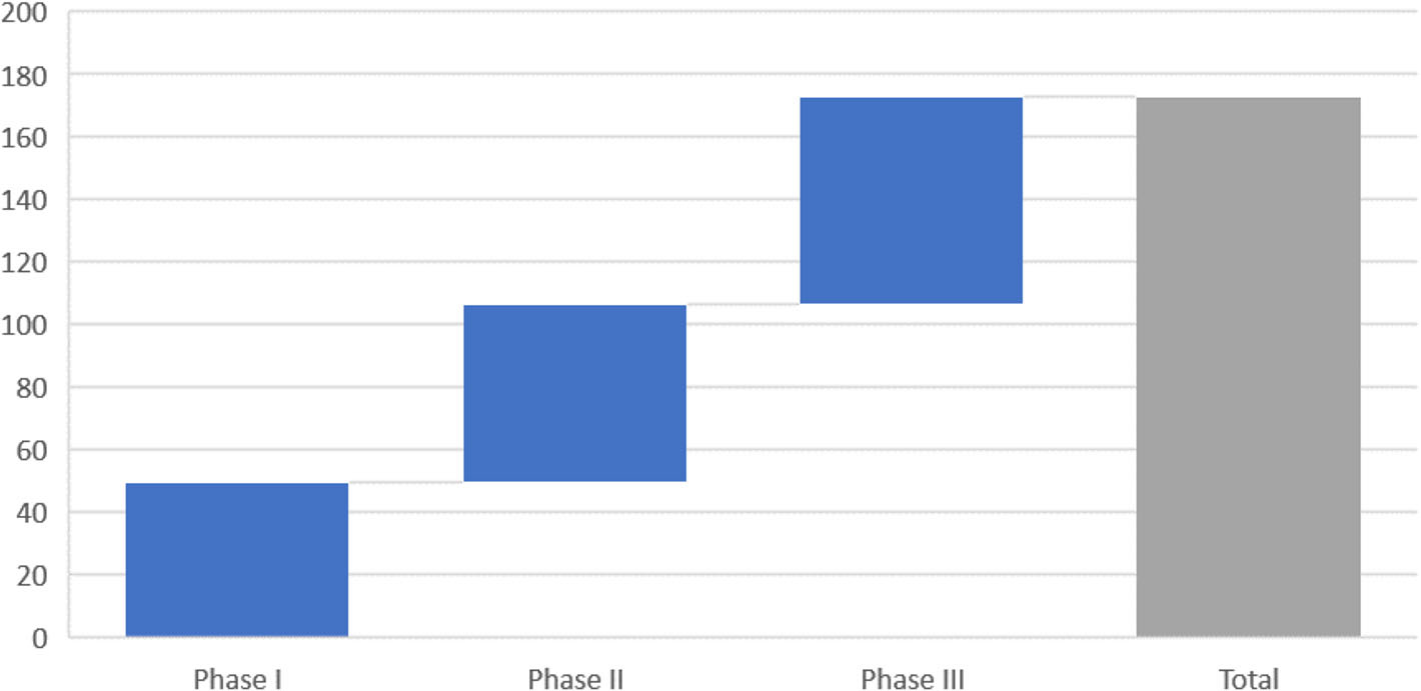
Expected phase specific costs in million USD based on DiMasi et al. (2016)

How do transition probabilities relate to R&D costs? There are several studies that estimate the cost of bringing a drug to the market [24, 25]. The latest is by DiMasi and co-authors and estimates the capitalised R&D costs to be 2558 million USD (in 2013 USD) of which 1460 million USD are attributed to the clinical stage and the remaining part to the pre-clinical stage. Capital costs are assumed to be 10.5% in their analysis. Capitalized expected phase specific costs are displayed in Fig. 3.

To calculate the cost of an approved drug, the transition probabilities between the different clinical phases are crucial input parameters. The lower the probabilities (i.e. the higher attrition rates), the higher the number of compounds a company will need to test in order to bring a single one to the market. This in turn would increase the R&D costs. DiMasi’s total clinical success rate of 11.83% means that a company would have to kick off 8,45 clinical development (1:0,1183) programs to bring one successful compound to the market. From a cost perspective, total expected capitalized development costs of 172.7 USD (Fig. 3) need to be multiplied with 8.45 to come up with DiMasi’s cost estimate of 1460 mill USD for the clinical development phase per approved drug.

### Costs of Pharmaceutical R&D and innovation

A few studies evaluate the effect of policy measures that reduce the cost of R&D [26]. Most of those studies examine R&D subsidies such as tax credits or tax deductions to determine if public money crowds out private investments or can trigger additional investments. Usually, if the price of an input (e.g. due to subsidies) goes down, the cost of producing the good goes down as well, and the supply of the good increases. We will utilise a study that estimates that a tax credit of 1 USD (which can be regarded as cost reduction by 1 USD) generates additional R&D expenditures of 0.293 USD [27]. This study is already from 1993, however, to the best of our knowledge, more up to date estimates are not available. To calculate absolute global values, we place our trial-specific estimates in relation to the global amount of business enterprise expenditures for pharmaceutical R&D [28]. To accommodate uncertainties around the value of the multiplicator of 0.293 we also check the robustness of the results by means of a sensitivity analysis. For this, we use the value of 0.293 as a base case. As a lower bound we report the results for halving the number (0.147) and as an upper bound by doubling it (0.586).

### Pharmaceutical R&D expenditures and gain in life-years generated

In a final step we will try to assess the effects that additional R&D expenditures have on outcomes in terms of number of life-years. For this purpose, we draw on the paper by Lichtenberg who analyzed all new molecular entities (NMEs) approved by the FDA during the time period 1950–1999 [29]. Lichtenberg estimates that private pharmaceutical R&D investments of 926 USD can generate a gain of one life-year in the US alone [29]. Adjusting these figures based on the US consumer price index (CPI) [30] to the 2013 prices used by DiMasi et al. yields an amount of 1218 USD.

## Results

**Fig. 4:**
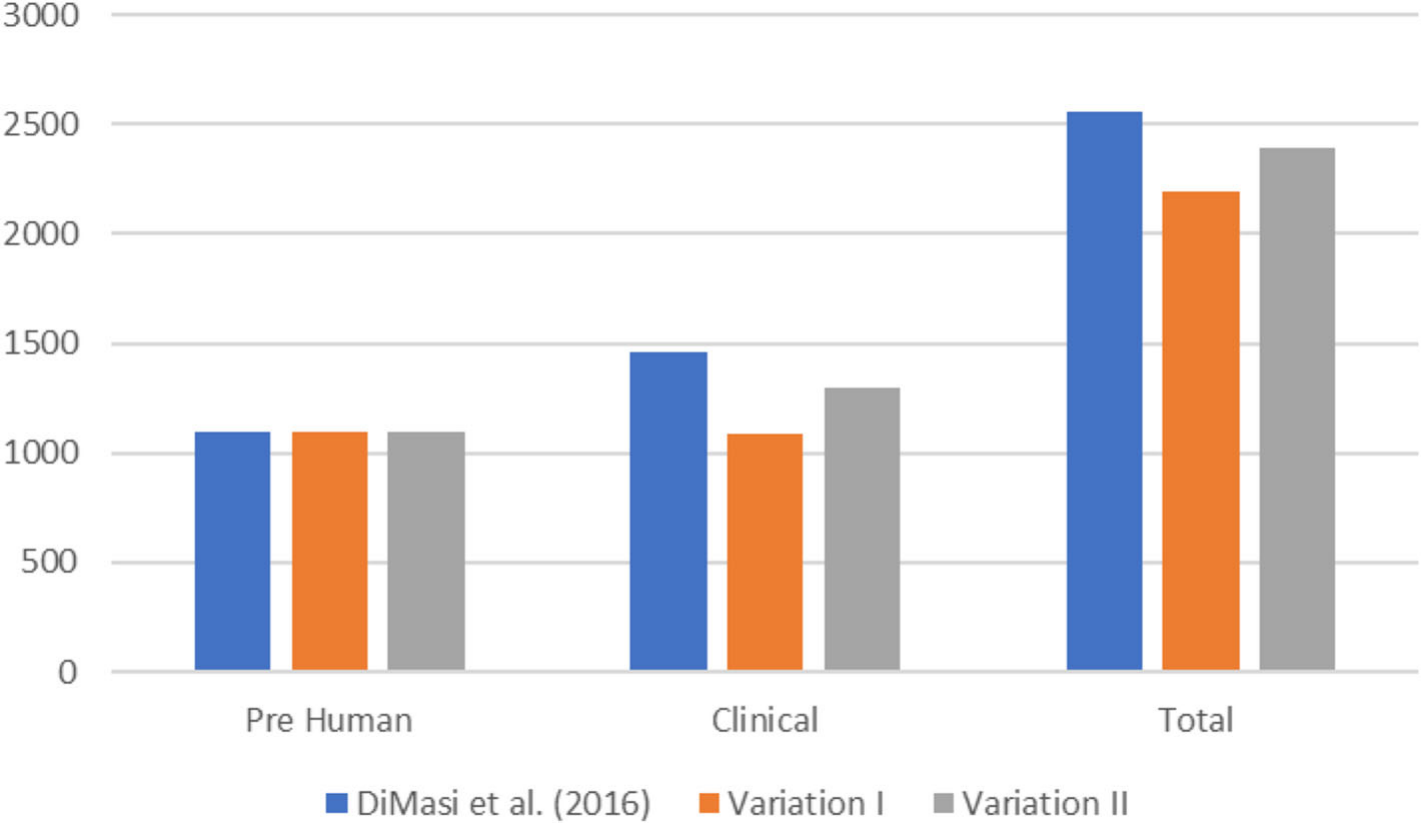
Cost estimates in million USD

Based on the assumptions stated above, the increase in clinical success rates would reduce the total costs of bringing a new drug to the market from 2.56 bn USD to 2.19 bn USD (− 14.4%) (variation I) and 2.39 bn USD (− 6.6%) (variation II), which is the result of a 25% (from 1.5 bn USD to 1.1 bn USD) and a 11.5% (from 1.5 bn USD to 1.3 bn USD) decrease, respectively, or 1.3bn USD) of the clinical phase (Fig. 4).

The cascade of economic implications is depicted in Table 1. The OECD estimates the total pharmaceutical R&D expenditures by business enterprises to be 100 bn USD. On a global scale, 14.4% (6.6%) cost reduction would translate into 14.4 bn USD (6.6 bn USD) in absolute values. It is a reasonable assumption that this cost decline could trigger demand for clinical research of additional 4.22 bn USD (14.4 bn USD × 0.29 USD) and 1.94 bn USD (6.6 bn USD × 0,29 USD) respectively. The respective confidence intervals using the values from the sensitivity analysis are [2.12 bn USD;8.44 bn USD] in variation I and [0.970 bn USD; 3.87 bn USD] in variation II[;Given Lichtenberg’s figures on the relationship between pharmaceutical R&D investments and the number of life-years gained, those additional 4.2 bn USD of R&D could save between 1.58 (1.94 bn USD: 1218 USD) (variation II) and 3.46 million life-years (variation I) in the US alone (4.2 bn USD: 1218 USD) with confidence levels between 1.3 mill and 6.9 million years.

**Table 1:** Impact Analysis

Impact	Variation of phase III transition probabilities	
	I (80%)	II (70%)
Overall trial success probability across all clinical phases	15.81%	13.37%
Cost per successful new drug	2190 million USD	2390 million USD
Cost savings per successful new drug compared to DiMasi et al. (2016)	370 million USD (−14.4%)	170 million USD (−6.6%)
Global cost reductions	14.4 bn USD	6.6 bn USD
Induced additional global R&D investments [lower; upper bound]	4.22 bn USD [2.12 bn;8.44 bn]	1.94 bn USD [0.970 bn; 3.87 bn]
Generated gain in life-years (in US) [lower; upper bound]	3.46 mill [1.74 mill; 6.93 mill]	1.58 mill [1.30 mill;3.17 mill]

## Discussion

We argue that it is reasonable to assume that a broader acceptance of adaptive trial design could improve R&D productivity by lowering attrition rates of phase III trials. If attrition rates could be reduced from 38 to 20%, overall clinical success rates would leap from 11.8 to 15.8%. This would reduce the costs per new drug by 14%. As a consequence, additional R&D investments of up to 4.2 bn USD could be encouraged, which, eventually, could generate a gain of up to 3.6 million life years by means of new drug launches. We believe that our estimates are conservative because we did not consider efficiency gains that occur in earlier clinical phases. As argued by the FDA, a design with adaptive dose selections may yield better estimates of the dose-response relationship, which is typically examined in phase II. This may also lead to more efficient trials later [13]. However, in our study we only described how adaptive designs would impact attrition rates in phase III. In addition, we extrapolated from other beneficial adaptive trial characteristics that might help to improve economic efficiency. For instance, adaptive clinical designs are better equipped to identify ineffective treatments sooner and thereby improve the allocation of resources for research and development [31]. For this reason, our cost estimates might even underestimate real productivity gains. On the other hand, there are also potential limitations of adaptive studies and thus potential cost drivers, such as the need for specific and complex analytical methods, a possible increased maximum patient sample size compared to a comparable fixed clinical study design, longer lead times between planning and initiation of the clinical trial and logistical challenges, e.g. to ensure high-quality interim data so that adaptive decision making is based on up-to-date and reliable results [13]. Although regulatory agencies such as the FDA are increasingly aware of the economic dimension of regulatory standards, global alignment with other regulatory agencies as well as with (mainly European) HTA bodies is necessary to fully reap the economic benefits. For instance, findings suggest that accelerated regulatory approval does not necessarily guarantee early market access because HTA bodies are not aligned with regulatory agencies [32]. Even now, pharmaceutical firms with a higher market presence in Europe invest less in R&D which can be lead to the conclusion that the highly regulated European drug markets reduce the companies’ incentives to invest in R&D [33, 34].

Of course, the wider acceptance of new clinical trial designs is not the only way to promote R&D productivity. Ideally it is complemented by innovative regulatory frameworks. An emerging problem is that private research investments are increasingly funnelled away from long-term projects that target diseases at an early stage or even before they develop [35]. For instance, there was recently a discussion of approaches that intercept the disease by delaying, stopping or reversing the causing pathophysiological process through targeted interventions before symptomatic disease develops. This approach, called “disease interception”, could be useful for targeting diseases such as cancer, Alzheimer’s (AD) or cardiovascular disease [36]. An increasing problem is that research of the early stages of these diseases is needed but financially not attractive because long-term clinical trials are required to detect relevant effects. Long-term trials do not only require costly investments. More importantly, the time span to recoup those investment costs is also much shorter because patent protection has effectively been reduced. For example, AD may well be suitable for disease interception due to its long preclinical phase characterised by pathophysiological processes which start many years before a patient develops the first clinical symptoms of AD [37]. Although research into Alzheimer’s disease has been ongoing for decades, there are only four approved drugs for symptomatic treatment whereas several compounds failed during clinical trials [38]. Potential policy instruments to remedy these problems are: acceptance of surrogate endpoints; direct R&D subsidies or an extension of patent protection [35].

In this context, it has long been argued that the drug approval process should be more continuous, so that patients have quicker access to beneficial drugs and there are more incentives to mount long-term studies, none of which the current system permits [39]. Manski [39] defines two error types in the drug approval decision. A type I error is when a new drug is approved based on preliminary data although it is actually inferior to the comparator. A type II error happens when better drugs do not receive approval because at the time of drug assessment, the data are still immature and do not prove superiority. Manski found that type II errors are more common when approval decisions are discontinuous. To reduce such inefficiencies, an innovative regulatory framework is called for that is not only effective in providing fast access but also in encouraging investment into early-stage disease research.

This issue gest addressed by EMA’s the adaptive pathway initiative [40]. Adaptive pathways are based on three principles: one is an iterative development by approval in stages (e.g. conditional approval) and confirming the benefit-risk ratio of a medical drug based on early clinical data using surrogate endpoints [16]. The inclusion of surrogate endpoints led to considerable criticism from various stakeholders [41, 42]. The EMA responded to this criticism by stating that some surrogate outcomes translated into favourable clinical outcomes while others did not, but that the issue is not specific for adaptive pathways [43]. Support for the EMA approach comes from the European Cancer Patient Coalition (ECPC) – calling it “the only potential life-saving procedure for patients suffering from rare and ultra-rare cancers”- representing over 400 cancer patient groups in 46 countries [44]. However, the approach is strongly refused by payers from various countries as they fear an increase in the uncertainty that patients would have to accept, resulting in a shift of risk from industry to patients [45]. The rejection is also justified by the difficulty of recruiting patients for post-approval studies conducted to obtain further safety and efficacy data [42]. The lack of a concept for generating real-world data after drug approval in order to draw robust conclusions about benefit and harm is also viewed critically [46]. Although not necessarily connected conceptually, adaptive trials designs are well suited for adaptive pathways since they may be used to optimize sample size, trial duration, and dose selection and reduce the negative side-effects of long classical RCTs like loss of subjects, placebo responses, and life-events [38, 47]. Therefore, an adaptive trial design can provide higher statistical efficiency to detect a true drug effect, or, alternatively, provide the same statistical power with a smaller expected sample size as a comparable non-adaptive design [13]. The advantages of ethical considerations should also be emphasized, because adaptive designs permit stopping a trial early if it becomes clear that the trial will be unlikely to demonstrate effectiveness [13].

Regulatory innovations such as adaptive pathways always need to balance economic perspectives with those of fast access and patient safety. Regarding safety, analyses in Canada and the USA have shown that serious adverse reaction warnings are more likely to be issued for drugs with conditional or accelerated approval than for drugs with regular approval [48]. This is in line with a finding that faster FDA approval has also seen higher numbers of black-box warnings and market withdrawals [49]. Also, Olson’s (2004) study suggests that shorter regulatory review times are associated with a higher rate of adverse events [50]. Conversely, a retrospective cohort study by Arnardottir et al. does not show any significantly increased safety risks for new drugs intended for the treatment of diseases with a “high unmet need”, which were granted accelerated approval in Europe [51]. On the other hand, while regulation can potentially reduce type I errors, the cost of regulation can be substantial as well. Several empirical economists studied the impact of the “Kefauvear-Harris Act “of 1962 that tightened regulatory approval. Their research results suggest that the regulation resulted in a stark decline in R&D productivity ranging between 60 and 80% and, as a result, in a decrease of new drug launches [52–54]. Of note, Grabowski and Vernon [55] conclude that “the hypothesis that the observed decline in new product introductions has largely been concentrated in marginal or ineffective drugs is not generally supported by empirical analyses”.

In general, our results contribute to the overarching debate on the relation between regulation and innovation. Although there is a controversy about the impact of regulation on innovation, many economists would agree that regulation hinders innovation rather than encourage it [56]. Blind et al. found that market uncertainty such as technological complexity is an important moderator in this context. In case of high market uncertainty, regulation leads to lower innovation efficiency (measured as innovation cost per employee) and vice versa [57]. On a policy level, the EU as well as other industrialised regions emphasise that the existing regulatory framework should be designed to support private-sector innovation activities [58, 59]. For this purpose, the EU commission has launched a “Pharmaceutical Strategy - Timely patient access” to strengthen the industry by creating a future-proof regulatory framework for the promotion of research and development of new medical products and technologies that meet the treatment needs of patients. In the view of the EU Commission the timely access of EU patients to state-of-the-art products may be hampered by the regulatory framework which is not fully adjusted for the use of real-world data and complex clinical trials for obtaining marketing authorization for medicines [60].

While we acknowledge some uncertainties concerning our estimations, we believe that the direction of the simulated effects is plausible. However, a full-fledged assessment of the impact of adaptive trials on R&D performance is beyond the scope of this study and would require more fine-grained data. Specifically, we have only looked at the association between cost reductions and the level of subsequent R&D investments. Other effects such as the potential impact on profit margins were beyond the scope of our analysis. Therefore, this is left for future research. Moreover, we acknowledge that there is some discussion about the link between R&D investments and life expectancy. Some authors such as Light and Lexchin [61] claim that most R&D investments are channeled into low risk R&D programs that provide only minor clinical advantages over existing treatments and therefore have only marginal impact on life expectancy. The impact on life expectancy depends mainly on drugs targeting severe and life-threatening diseases. The basis of our investigation of the extent to which additional R&D spending affects the number of life years is the work of Lichtenberg [29]. The strength of this study is that Lichtenberg counted unselected, every FDA-approved NMEs in the period 1950–1999. Accordingly, the analysis includes NMEs for the treatment of mild and treatable diseases as well as life-threatening and fatal diseases. Subsequent extensions of approval in new therapeutic areas are not included in the study, which is certainly a weakness of the study. But in the overall assessment of the Lichtenberg methodic, we do not see the risk of overestimating the observed increase in life years per additional R&D expenditure.

Finally, there are studies in the literature that evaluate new drug approvals from the FDA or EMA with regard to the underlying therapeutic areas. At least in the recent past (the period of investigation of the respective studies is 2000–2017 and 2014–2016), the majority of new approvals are found in the therapeutic areas e.g. of oncology, neurology, infectious diseases and cardiovascular diseases [62, 63]. This trend in drug approvals supports our approach and results to which extent R&D spending results in increased life years.

## Conclusion

In order to ensure a continued stream of innovative new drugs and to achieve leap innovations in therapeutic areas in the future, it is essential to improve the economic efficiency of clinical trials. New clinical trial designs such as adaptive designs going hand in hand with regulatory innovations are key to increasing productivity in the pharmaceutical industry. Such productivity increases could not only provide new R&D investment incentives; they would also mitigate affordability issues and remedy the observed “cost disease” of advanced health-care systems. Therefore, measures to promote a sustainable health-care system should not only be considered from the end of the value chain (e.g. price regulations), but first of all from its beginning (e.g. R&D investment incentives).
